# Mob4 is required for neurodevelopment in zebrafish

**DOI:** 10.17912/micropub.biology.000762

**Published:** 2023-02-24

**Authors:** Claudia Florindo, Jorge M. Mimoso, Sandra L. Palma, Carolina Gonçalves, Daniela Silvestre, Marco Campinho, Álvaro A Tavares

**Affiliations:** 1 Faculty of Medicine and Biomedical Sciences. University of Algarve; 2 Centre for Biomedical Research, University of Algarve, 8005-139 Faro, Portugal.; 3 Present address: Andor Technology, Belfast, UK.; 4 Present address: Centro Hospitalar Universitário do Algarve, Faro, Portugal; 5 Present address: Hospital Garcia de Orta, Serviço Neurologia, Almada, Portugal.; 6 Algarve Biomedical Center (ABC), University of Algarve, 8005-139 Faro, Portugal; 7 Marine Science Centre (CCMAR), Universidade do Algarve; 8 Algarve Biomedical Center-Research Institute (ABC-RI)

## Abstract

Mob4 is an essential evolutionary conserved protein shown to play roles in cell division and neural development. Mob4 is a core component of the macromolecular STRIPAK complex involved in various critical cellular processes, from cell division to signal transduction pathways. However, Mob4 remains relatively poorly understood. Although the consequences of eliminating Mob4 function in Drosophila are described, its function in vertebrate development remains largely unknown. Here we show that knockdown and knockout of Mob4 during zebrafish embryogenesis limits neuronal cell divisions but has little effect on apoptosis, thus arguing a role for mob4 in neurodevelopment.

**Figure 1. Downregulationof Mob4 in early embryos affects cell proliferation and neurodevelopment.**
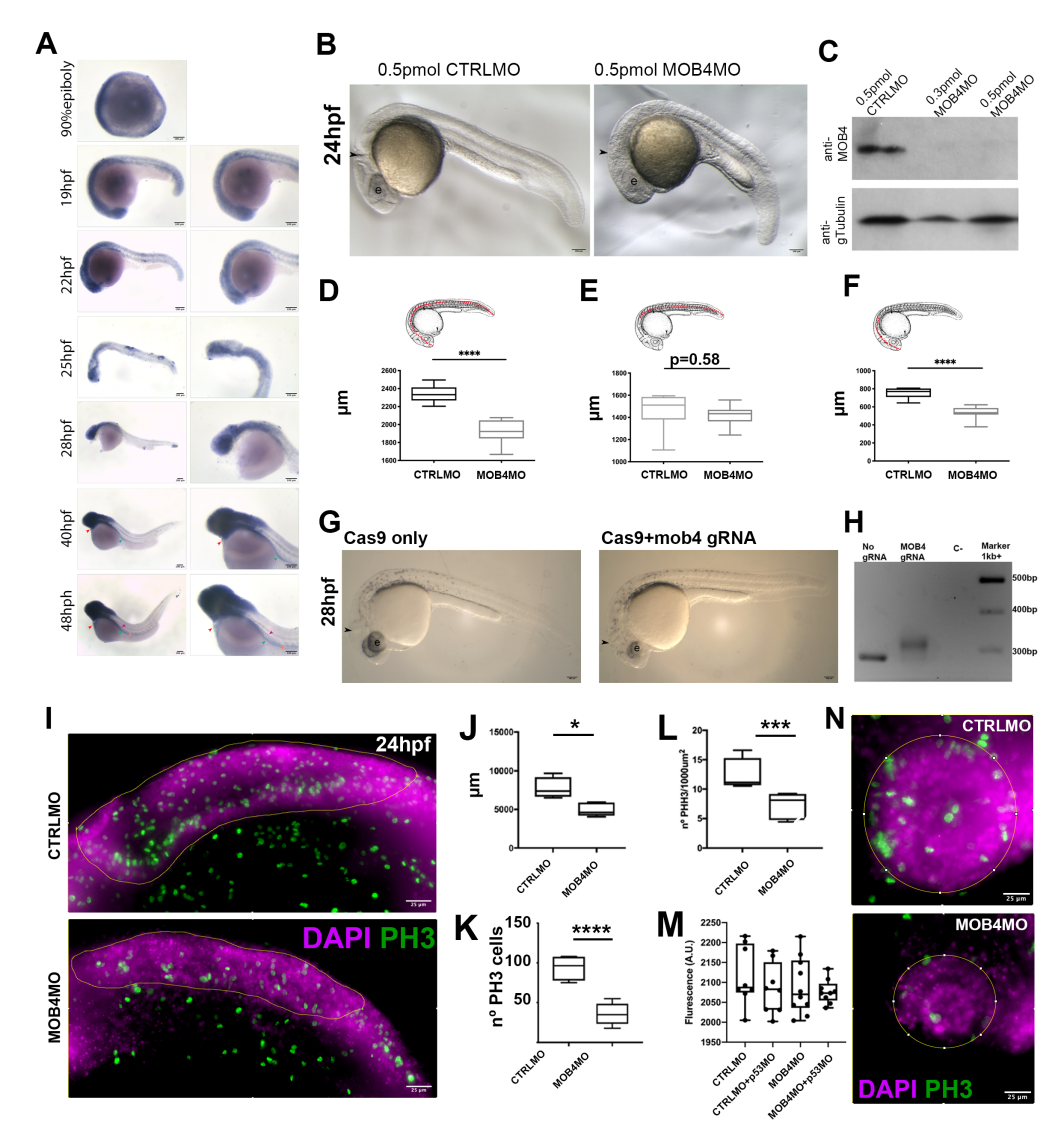
(A) In situ hybridization expression analysis of zebrafish mob4 in wild-type embryos from 90% epiboly, 19, 22, 25, 28, 40, and 48hpf. Bars represent 100 mm. Morpholino-mediated knockdown (KD) of zebrafish mob4 leads to severe neurologic defects during embryogenesis. (B) At 24hpf, morpholino KD of mob4 gives rise to severe neurologic defects. The MHB (arrowhead) is almost absent in 0.5pmol MOB4MO injected embryos, and the eye (e) is smaller. Western blot (C) confirms depletion of Mob4 protein in 24hpf embryos injected with 0.3 and 0.5pmol of MOB4MO but not in 0.5pmol CTRLMO injected siblings. The loading control was carried out using an anti-gamma tubulin antibody (GTU-88). Standard length (D) of 0.5pmol MOB4MO injected embryos is smaller than CTRLMO injected siblings. Trunk length (E) is not affected by 0.5pmol MOB4MO injection. Head length (F) is significantly smaller in 0.5pmol MOB4MO injected embryos. T-test, **** p<0.0001. Bars in A represent 100mm. MOB4 knockout (KO) in CRISPR/Cas9-generated crispants fully recapitulates the MOB4 morpholino phenotype. In no gRNA, Cas9 only, injected F0 embryos (crispants) present a normal phenotype (G) for at 28hpf whereas embryos injected with a gRNA aimed at exon 1 of zebrafish MOB4 gene locus present severe impairment of neural structures. In MOB4 crispants the MHB and the eye (e) are underdeveloped with the phenotype reminiscent of the MOB4MO injected siblings. PCR genotyping (H) confirmed the introduction of an insertion on the zebrafish MOB4 locus in F0 crispants. MOB4 is involved in hindbrain cell proliferation during zebrafish neurodevelopment. (I) Maximum projections of Z-stacks of the hindbrain (yellow line delimited region) of 24hpf embryos where labelling of mitotic cells after PH3 (green) and DAPI (magenta) immunohistochemistry reveals that after injection of 0.5pmol MOB4MO leads to reduced hindbrain area (J) and cell divisions number (K and L, t-test: *p<0.05, ***p<0.003, ****p<0.0002). On the other hand, TUNNEL analysis followed by whole-embryo fluorescent analysis (M) does not indicate significant differences in apoptotic cell number between 0.5pmol CTRLMO and MOB4MO injected embryos. Moreover, co-injection with 0.5pmol p53MO cannot change apoptosis indexes in any experimental group (one-way ANOVA, p>0.05). (N) Maximum projection images of z-stacks of the eye (yellow line delimited) of CTRL and MOB4MO injected embryos labeled with PH3 (green) and DAPI (magenta) show a decrease in mitotic cell number.

## Description

The striatin-interacting phosphatase and kinase (STRIPAK) complex is a mediator and regulator of multiple vital signalling pathways, including Hippo, MAPK (mitogen-activated protein kinase), nuclear receptor and cytoskeleton remodelling. The complex is evolutionary, highly conserved from lower to higher eukaryotes, and contains kinase and phosphatase subunits. The human STRIPAK complex is a large, multisubunit PP2A complex with striatin as the regulatory subunit. The core STRIPAK contains, in addition to the PP2A catalytic (PP2Ac) and scaffolding (PP2A A) subunits and striatins, including STRN-interacting protein 1 or 2 (STRIP1/ STRIP2) and MOB4. Several other proteins may associate with the core complex, conferring different biological functions (Kück, et al. 2019). Different STRIPAK complexes are associated with fundamental biological processes ranging from cell growth, differentiation, proliferation and apoptosis to metabolism, immune regulation and tumorigenesis (Shi, et al. 2016).

MOB4, a core component of STRIPAK in Drosophila and humans, belongs to the Mob protein family, a family of adaptor proteins that regulate kinase activities. In human cells, MOB4 can form a complex with MST4 kinase and disrupt MST1-MOB1 complex and increases YAP1 activity, the endpoint of the Hippo cascade, implying that MOB4 can be oncogenic (Chen et al., 2018). In planaria, mob4 restricts posterior body length by inhibiting the wnt signalling in neuroblasts (Schad and Petersen, et al. 2020). In Drosophila, Mob4 is an essential developmental gene and has been shown to associate with microtubules to regulate neuronal morphogenesis. In zebrafish, mob4 mutants are associated with impaired skeletal muscle sarcomere assembly and muscle development (Berger, et al. 2022). However, little is known about the role of mob4 in vertebrate neurodevelopment. Here we address the consequences of the downregulation of Mob4 in the initial stages of zebrafish embryogenesis.

Expression of zebrafish MOB4 was determined by in situ hybridization in wild-type embryos from at least 90% epiboly to 48hpf. The zebrafish genome encodes only one mob4 homolog. Expression is observed in the full extension of the neuroectoderm at 90% epiboly (Fig. 1A). At 19 hours post-fertilization (hpf), expression is more robust in the brain. However, patches of expression are also observed in the somites (Fig. 1A). That pattern of expression continues as development progresses but becomes more circumscribed to the brain from 22hpf onwards (Fig. 1A). At 28hpf, expression of mob4 is mainly on the brain with small patches of expression in the most anterior somites (Fig. 1A). By 40hpf there is no expression on the somites and a strong expression is now found in the brain and kidney primordium (green arrowhead in Fig. 1A). In the heart is also found expression of mob4 (red arrowhead in Fig.1A). Just before hatching, at 48hpf, mob4 is almost exclusively expressed in the brain (Fig. 1) but also in the kidney primordium (green arrowheads in Fig. 1A). Some scattered expression in the heart expression is found (red arrowheads) but expression has reduced from 40hpf (Fig. 1A). However, some patches of expression are now observed in the dorsal aorta and the caudal vein (respectively magenta and orange arrowheads in Fig. 1A). The expression of mob4 during embryogenesis supports previous reports on skeletal muscle development (Berger, et al. 2022). This evidence shows that mob4 must have a role in zebrafish neurodevelopment.

**Knockdown of mob4 leads to severe neurologic defects.** The function of MOB4 was investigated by a knockdown (KD) approach using translation-blocking morpholinos (MO) against the Kozak sequence of the zebrafish mob4 gene (Fig. 1B). This approach altogether abolished Mob4 expression at 0.5pmol as confirmed by western blot (Fig. 1C). Zebrafish embryos at 24hpf presented severe neurologic defects as observed by the lack of midbrain-hindbrain boundary (MHB, arrowhead in Fig. 1B) and reduced eye size (e in Fig. 1B). A quantitative analysis on the morphological consequences of mob4 KD revealed that mob4 morphants are significantly smaller than their control morpholino siblings (t-test p<0.0001, Fig. 1D). Notably, these differences are not found in the axial trunk (t-test p>0.05, Fig. 1E) but only in the head (t-test p<0.0001, Fig. 1F). This evidence strongly supports that MOB4 action is related to neurodevelopment of the brain but not the spinal cord.

We further validated our findings on mob4 function in zebrafish embryogenesis by CRISPR/Cas9-based knockout of mob4 (Fig. 1G). Using this approach, where a gRNA was used to target the Kozak sequence of the zebrafish mob4 gene, crispant embryos (F0 injected embryos) revealed an identical phenotype to the one using the translation blocking MO (Fig. 1B and G). In the mob4 crispant embryos, as in the mob4 morphants, the MHB does not develop, and eye development is severely compromised, thus presenting a smaller size (e in Fig. 1B and G). Further genotyping of these mob4 crispant embryos confirmed the introduction of genetic lesions on the mob4 locus (Fig. 1H). The obtained results with mob4 crispants confirm the neurodevelopmental effects in zebrafish mob4 morphants (Fig. 1B and G).

Using the mob4 KD approach, it was found that in these embryos, cell divisions in the hindbrain and eye are severely diminished (Fig. 1I-K and N). As previously seen (Fig. 1F), the brain, particularly the hindbrain, is especially dependent on mob4 function for development. A more detailed analysis reveals that the hindbrain is significantly smaller in mob4 morphants (yellow delimited area in Fig. 1I and J, t-test p=0.02). Moreover, as denoted by Phospho-histone H3 staining (PH3), the number of mitotic cells in the hindbrain is significantly reduced (Fig. 1I and K, t-test p=0.0002). Even if PH3 staining is considered a function of the overall hindbrain area, the number of mitotic cells continues to be reduced (Fig. 4L, t-test p=0.003). These findings argue that reduced hindbrain size is due to impaired cell division.

Notably, we do not detect any increase in whole-embryo apoptosis, after TUNNEL assay and total fluorescent measurement at 570nm, in mob4 KD embryos when compared to control embryos (Fig. 4M, one-way ANOVA p>0.05), suggesting that reduction of the size of the hindbrain (and the brain) is likely due to impaired cell proliferation rather than increased cell death. Moreover, no MO produced any unspecific apoptosis given that co-injection of p53 MO (Robu, et al. 2007), which prevents p53 mediated apoptosis, did not reduce apoptosis compared to injection of CTRL and MOB4 MO (Fig. 1M). Notwithstanding, the total fluorescent analysis of the apoptosis carried out has limitations, as we cannot rule out tissue-specific apoptosis. That can only be confirmed by a detailed image analysis that was not carried out in this study.

In the eye, there was a severe decrease in mitotic cells and a decrease in organ size (Fig. 1N). Together with our previous results, this evidence strongly suggests that mob4 is involved in cell proliferation during neurodevelopment. Although we have not identified which cell types have affected proliferation, our data agree with the findings in planaria, where mob4 regulates cell proliferation of progenitor cells (Schad and Petersen, et al. 2020).

In conclusion, our results indicate that mob4 is involved in zebrafish embryogenesis, involved in cell proliferation, but not survival, of progenitor cells, a function that is likely to be conserved from planaria to mammals.

## Methods

*In situ hybridization (ISH):* Wild-type AB strain male and female adult animals were separated in mating boxes overnight and kept at 14h:10h light: dark at 28C. The following morning the separation on the maiting boxes was taken out, and the animals were allowed to spawn naturally. Embryos were collected to E3 medium (Campinho, et al. 2014) and kept at 28.5C until sampling. At 90% epiboly, 19, 22, 25, 28, 40 and 48 hpf embryos, according to morphological landmarks by Kimmel (Kimmel, et al. 1995), were decorinated manually and fixed overnight in 4%PFA/1xPBS (pH 7.4) at 4C. In the following morning, embryos were rinsed, at room temperature (RT), in 1xPBS+0.1% Tween-20 (Sigma; PTW) several times, bleached in 1xPBS/0.5%KOH/3%H2O2 and transferred to 100% Methanol (MeOH), through a graded series of PBS/MeOH, and kept at -20C until use. ISH was carried out as described previously (Campinho, et al. 2014). Photos were taken in an Olympus SZX7 stereoscope coupled to a digital colour camera (Optika) and analyzed in FIJI(Schindelin, et al. 2012).

*mob4 knockdown assay:* 1-cell stage embryos were collected after natural spawning and injected with 0.3 or 0.5pmol of translation blocking mob4 morpholino (CCTCCGCCATGACCATCTCGACAGA) (n≥500/experimental group; SEQ; Gene Tools). The standard control MO (Gene Tools) was injected at 0.5pmol to dismiss any non-specific effects. After injection, the embryos were incubated at 28.5C in E3 medium. At 24hpf, embryos were dechorionated, anesthetized with 0.16% MS222 in E3, and imaged (n≥20/experimental group) in a Zeiss Stemio 305 stereoscope coupled to a digital camera (Optika). Three pools of 100-200 24hpf embryos per experimental group were dechorionated and collected for whole-protein extraction using already established methods (Link, et al. 2006). Western-blot (WB) to detect zebrafish Mob4 was carried out using the anti-human Mob4 antibody 16E2 (Santa Cruz, sc-80664), at 1:1000.

*Mob4 knockout assay:* Wild-type embryos were generated by natural spawning, as described previously. 1-cell stage embryos were injected with 600pg Cas9 protein (Champalimaud Foundation) with 300pg of in vitro prepared guide RNA (gRNA) against a target sequence in the first exon of mob4 (GGCGAGATGGTCATGGCGGA). After injection, embryos were incubated at 28.5C in E3 medium. Control embryos were injected with 600pg of Cas9 protein only. The gRNA was designed against mob4 locus using the CRISPRScan software, chosen after the determination of no hits besides the mob4 exon 1 locus and prepared accordingly to (Moreno-Mateos, et al. 2015). Injected embryos (F0) were sorted for completely penetrant phenotype, decorinated, anaesthetized with 0.16% MS222 (Sigma) and transferred to 3%Mehylcellulose/1xE3/0.016%MS222 for photographic registration (n>10/group) at 28hpf. Imaged embryos were collected, and genomic DNA was extracted. Genomic DNA extraction was carried out after incubation overnight at 50C in 50uL of 100mM Tris-HCl, 50mM MgCl _2_ , 100mM NaCl, 0.1% Tween20 and 200ng/uL of proteinase K (Carl Roth). The following morning homogenized embryos were added 100uL of 100% ethanol (EtOH) and centrifuged at 4C for 30 minutes at 14000rpm. The supernatant was discarded, and genomic DNA was washed with 1mL of ice-cold 70%EtOH and centrifuged at 4C for 5 minutes at 14000rpm. The supernatant was discarded, and the pellets were allowed to dry and resuspended in 50uL of TE. PCR against the zebrafish mob4 exon 1 was carried out with primers (Forward: GATAACACTCGATGTAAGATGG; Reverse: TAGTACATTAGGATGTGCTCC) flanking the gRNA target sequence. PCR reactions were carried out in 1x DreamTaq Buffer, 0.2nM of primers, 0.2mM dNTPs and 0.06U/uL of DreamTaq (ThermoFisher). Thermocycle was as follows: 5 minutes at 95C, 35 cycles of 30 seconds at 95C, 20 seconds at 58C and 20 seconds at 72C, followed by a final step of 5 minutes at 72C. PCR products were separated in a 3% agarose/1xTAE gel after collecting electrophoresis gel images.

*Immunohistochemistry (IHC):* Embryos for IHC were previously injected with control and mo4 Mos as described above. IHC was carried out overnight at 4C in 1xPBS/0.5% Triton-X (Carl Roth) using 1/400 rabbit anti-phosphatated Histone 3 (PH3; Sigma) antibody followed by incubation with 1/6goat-anti-rabbitbit IgG (H+L) serum conjugated with CF488 (Sigma) followed by 1h in PBS with 1/20000 DAPI (Carl Roth). At 24hpf, embryos were fixed in 4%PFA/1xPBS as described previously. Embryos were mounted, and Z-stacks were taken from the hindbrain and eye in a Zeiss Z2 upright widefield fluorescent microscope coupled to a CoolPix digital camera (n≥5). Images were transferred to FIJI to determine the number of PH3+ cells in the hindbrain manually.

*Apoptosis TUNNEL assay:* For TUNNEL apoptosis assay, 1-cell stage embryos were injected with 0.5pmol CTRMO, 0.5pmol MOB4MO, 0.5pmol CTRMO+0.5pmol p53MO (p53 morpholino - GCGCCATTGCTTTGCAAGAATTG) (Robu, et al. 2007) or 0.5pmol MOB4MO+0.5pmol p53MO. This approach allows us to determine if there is non-specific apoptosis due to the non-specific effects of CTR and MOB4Mos. Embryos were fixed in 4%PFA/1xPBS at 24hpd, as described above. TMR red TUNNEL apoptosis assay (Roche) was carried out accordingly to the manufacturer's instructions (n≥5/per group). Individual embryos were transferred to a BD Falcon 96 Flat Bottom Black Polystyrene 96-well plate with 100uL of PBS, and whole fluorescent was measured in a Tecan Infinite plate spectrophotometer. Embryos were excited to a 545nm wavelength, and fluorescence was measured at 570nm.

*Statistical analysis:* Unpaired Students t-test was used to determine differences between experimental groups after confirmation of normality by Bartlett's test. For apoptosis analysis, One-way ANOVA was carried out, and differences between experimental groups were determined after the Bonferroni post-hoc test. Statistical significance was considered if p≤0.05. All statistical analysis was carried out in GraphPad version 8.
